# The Potential Impact of the New York State Smokers’ Quitline on Population-Level Smoking Rates in New York

**DOI:** 10.3390/ijerph16224477

**Published:** 2019-11-14

**Authors:** Nathan Mann, James Nonnemaker, Kevin Davis, LeTonya Chapman, Jesse Thompson, Harlan R. Juster

**Affiliations:** 1 RTI International, Research Triangle Park, NC 27709, USA; jnonnemaker@rti.org (J.N.); kcdavis@rti.org (K.D.); lchapman@rti.org (L.C.); jmthompson@rti.org (J.T.); 2 Bureau of Tobacco Control, New York State Department of Health, Albany, NY 12242, USA; harlan.juster@health.ny.gov

**Keywords:** quitlines, population impact, tobacco use cessation, tobacco control

## Abstract

Receiving smoking cessation services from telephone quitlines significantly increases quit success compared with no intervention or other quitting methods. To affect population-level smoking, quitlines must provide a sufficient proportion of smokers with effective interventions. Nationally, quitlines reach around 1% of adult smokers annually. From 2011 through 2016, the average annual reach of the New York State Smokers’ Quitline (NYSSQL) was 2.9%. We used data on the reach and cessation outcomes of NYSSQL to estimate its current impact on population-level smoking prevalence and to estimate how much reach would have to increase to achieve population-level smoking prevalence reductions. We estimate NYSSQL is associated with a 0.02 to 0.04 percentage point reduction in smoking prevalence in New York annually. If NYSSQL achieved the recommended annual reach of 8% (CDC Best Practices) and 16% (NAQC), state-level prevalence would decrease by an estimated 0.07–0.12 and 0.13–0.24 percentage points per year, respectively. To achieve those recommended levels of reach, NYSSQL would need to provide services to approximately 3.5 to 6.9 times more smokers annually. Given their reach, quitlines are limited in their ability to affect population-level smoking. Increasing quitline reach may not be feasible and would likely be cost-prohibitive. It may be necessary to re-think the role of quitlines in tobacco control efforts. In New York, the quitline is being integrated into larger efforts to promote cessation through health systems change.

## 1. Introduction

All U.S. states maintain and operate state telephone tobacco cessation quitlines that offer free tobacco use cessation counseling and mailed self-help materials to tobacco users. Some state quitlines also offer free nicotine replacement therapy (NRT) and web-based cessation services. States implement and fund quitlines to increase tobacco use cessation and contribute to overall reductions in tobacco use by removing barriers to treatment. Tobacco quitlines have been shown to effectively increase tobacco use cessation and are a recommended component of comprehensive state tobacco control programs (TCPs) [1,2,3,4,5]. Quitline counseling significantly increases quit success compared with minimal intervention, self-help, or no counseling [5].

For tobacco quitlines to have a meaningful impact on tobacco use at the population level, they must be able to provide effective interventions to a sufficient proportion of tobacco users [6]. The potential impact of quitlines on population-level smoking rates depends on the quitline reach, defined as the proportion of smokers who receive a service from the quitline and effectiveness in helping smokers quit. The Centers for Disease Control and Prevention’s (CDC’s) 2014 *Best Practices for Comprehensive Tobacco Control Programs* recommends that state quitlines aim to reach approximately 8% of smokers annually [2]. In 2009, the North American Quitline Consortium (NAQC) estimated that, with promotions and sufficient funding, quitlines could potentially reach 16% of smokers annually [7]. From the early 2000s through to 2015, the annual reach for state quitlines in the United States averaged between 1% and 2% of all smokers, nationally [2,3,6,8,9,10,11,12,13,14,15]. Data from previous studies and from CDC’s National Quitline Data Warehouse (NQDW) indicate that the annual state-level quitline reach ranges from less than 0.1% to around 4.0% [14,15].

Reducing population-level tobacco use is a primary objective of state TCPs, who are the primary funders of state quitlines. The goals of the National Tobacco Control Program, which provides limited funding to all U.S. states, include promoting tobacco use cessation and reducing population-level tobacco use through population-based community interventions [16]. Other key federal guidelines, such as the 2014 CDC *Best Practices* and the 2014 U.S. Surgeon General’s report, stress the importance of using population-based efforts to reduce tobacco use at the population level. Maximizing quitline reach and using quitlines to reduce population-level smoking rates are goals clearly implied by CDC and NAQC emphasis and publications [2,7]. However, state TCPs use quitlines in varying ways. For some states, quitlines may be their primary or perhaps only cessation tool due to lack of funds or the belief that quitlines sufficiently facilitate population-level tobacco use cessation. Other states use quitlines as part of a larger, more comprehensive approach for increasing tobacco use cessation and reducing overall tobacco use at the population level [2,3]. From the perspective of state TCPs, understanding the role of quitlines in their larger efforts to reduce population-level tobacco use is important to consider.

New York implements a telephone quitline as one component of their larger, comprehensive state tobacco control program [17]. The New York State Smokers’ Quitline (NYSSQL), administered by Roswell Park Cancer Institute (RPCI) under contract to the New York State Department of Health (NYSDOH), has been in operation since 2000. NYSSQL provides free information, tools, quit coaching, and support to tobacco users in both English and Spanish and offers free NRT starter kits to eligible callers. NYSSQL also works with health care providers, health plans, and employers to ensure that tobacco users have access to effective cessation assistance and treatment. NYSSQL is one of the largest state tobacco cessation quitlines in the United States in terms of call volume and percentage of the state tobacco user population it reaches.

The purpose of this paper is to examine the potential impact of quitlines on population-level smoking rates. We use data from New York to address three unanswered research questions related to the potential population impact of quitlines: (1) what is the current impact of quitlines on population-level smoking prevalence?; (2) what is the potential impact of quitlines on population-level smoking prevalence if the quitline reach increased to recommended levels?; and (3) how much would it cost to increase annual quitline reach to recommended levels? We examine each of these research questions from the perspective of comprehensive state TCPs that implement and largely fund telephone quitlines as one component of a larger system of interventions designed to reduce population-level tobacco use.

## 2. Methods

### Data

We obtained quarterly data on quitline utilization from 2011 through 2016 from the CDC’s NQDW available on the CDC’s State Tobacco Activities Tracking and Evaluation (STATE) System website (https://www.cdc.gov/statesystem/). Quarterly quitline utilization measures include the total number of calls to the NYSSQL and the unique number of quitline callers who received counseling and/or free NRT from the NYSSQL. We created annual estimates of quitline call volume and callers who received counseling and/or free NRT by summing quarterly data by year. We obtained data on annual operational costs of the NYSSQL and the amount and costs of paid television ads purchased by NYSDOH to promote smoking cessation and the quitline telephone number for 2011 through 2015. We obtained annual estimates of the adult civilian population in New York from the U.S. Census Bureau based on the 2010 Census. We used annual estimates of the prevalence of current adult cigarette smoking in New York for 2011 through 2016 from CDC’s Behavioral Risk Factor Surveillance System (BRFSS). We estimated the total number of adult smokers in New York by multiplying Census population estimates of the number of adults in New York by the prevalence of current smoking from the BRFSS.

We obtained data on quitline effectiveness from 7-month follow-up evaluation surveys collected from June 2010 through December 2014 by RPCI. These surveys were administered to a random sample of NYSSQL callers approximately 7 months after receipt of NYSSQL services and include three different measures of quit success: (1) quit for 7 days or longer, (2) quit for 30 days or longer, and (3) quit for 6 months or longer. Each of these three quit rates was calculated using two different methods to provide upper- and lower-bound estimates: a follow-up survey responder rate and an intent-to-treat rate. Follow-up survey responder rates were calculated only among respondents who completed 7-month follow-up evaluation surveys. Intent-to-treat quit rates attempt to correct for the potential nonresponse bias of the 7-month follow-up evaluation survey. Intent-to-treat rates include individuals who completed 7-month follow-up evaluation surveys and those who were sampled for but did not complete 7-month follow-up evaluation surveys in the denominator. The numerator is the same for both the responder rate and intent-to-treat methods, but the denominators differ. The responder rate is likely to be an optimistic estimate because of the potential nonresponse bias of the follow-up evaluation survey, and the intent-to-treat rate is likely to be a conservative estimate because of the assumption that all nonresponders failed to make a quit attempt or maintain sustained quitting behaviors.

Research Question 1: What is the current impact of quitlines on population-level smoking prevalence?

We present annual trends in the total number of adult cigarette smokers in New York, calls to the NYSSQL, and number of tobacco users who received quitline services from 2011 through 2016. We also summarize the effectiveness of the NYSSQL by presenting 7-day, 30-day, and 6-month quit rates from the 7-month follow-up evaluation survey data. We present estimates separately for each of the quit rates based on the 7-month follow-up responder quit rates and the intent-to-treat quit rates. To estimate the number of successful quitters among tobacco users who received services from the NYSSQL, we multiplied the total unique number of quitline clients who received counseling and/or free NRT from the NYSSQL by the various quit rate measures from the 7-month follow-up evaluation survey data.

To estimate the current impact of the NYSSQL on population-level smoking rates in New York, we subtracted the number of quitline clients in 2016 who were smoke-free for 6 months or more at the 7-month follow-up from the total number of current adult cigarette smokers in New York. We then divided the resulting smoking population estimate by the total New York adult population to calculate the prevalence of adult cigarette smoking if the number of quitline clients in 2016 who were smoke-free at 7-month follow-up quit permanently and were removed from the adult smoking population. The NYSSQL’s population-level impact was calculated separately based on the 7-month follow-up survey responder rate and the intent-to-treat variations of the 6-month quit rate.

Research Question 2: What would be the impact on population-level smoking prevalence if quitline reach increased to recommended levels (CDC Best Practices for Comprehensive Tobacco Control, NAQC)?

CDC’s *Best Practices for Comprehensive Tobacco Control* recommends that state quitlines strive to reach 8% of states’ adult smoker population each year, whereas the NAQC indicates sufficiently funded quitlines should be able to annually reach 16% of adult smokers. We estimated the impact of the NYSSQL on population-level smoking prevalence if it achieved an annual quitline reach of 8% and 16%. We then compared the estimated population-level impact of the NYSSQL in these two scenarios to its estimated actual population-level impact in 2016.

To estimate the population-level impact of the NYSSQL in our two counterfactual scenarios, we first calculated the total number of adult smokers who would have received counseling and/or free medication from the NYSSQL. Based on the same quit rates used for our base scenario in our first research question, we estimated the number of quitters the quitline would generate as well as the state-level quit rate associated with quitline quitters. The formula used to estimate quit rates associated with quitline quitters in our two hypothetical reach scenarios is given by Equation (1):

(1) $$Quit\;Rate_{NY}=\frac{\left(Adult\;Smokers_{NY}\;\times \;Reach_{recommended}\right)\;\times \;Quit\;Rate_{6-month}}{Adult\;Smokers_{NY}}.$$

We multiplied the number of adult tobacco users the NYSSQL reached in 2016 by the 6-month quit rates to estimate the number of quitters the quitline could generate. We then divided the estimated number of quitline quitters by the current New York adult smoker population to estimate the state-level quit rate associated with quitline quitters.

When calculating New York’s smoking prevalence upon achieving the recommended reaches in our counterfactual scenarios, we accounted for the number of successful quitline quitters estimated using Equation (2). We assumed these quitters remained quit, removed them from the 2016 adult smoker population, and then re-calculated statewide smoking prevalence. Equation (2) shows the formula we used to estimate statewide smoking prevalence upon the NYSSQL achieving a reach of 8% and 16%:

(2) $$Prevalence=\left\{\frac{Adult\;Smokers_{NY}-\left[\left(Adult\;Smokers_{NY}\;\times \;Reach_{recommended}\right)\times Quit\;Rate_{6-month}\right]}{Adults_{NY}}\right\}.$$

Using the formula above, we calculated two smoking prevalence estimates for each counterfactual scenario—one estimate using the intent-to-treat quit rate and another estimate using the responder quit rate.

Research Question 3: How much would it cost to increase the annual quitline reach to recommended levels (CDC Best Practices for Comprehensive Tobacco Control, NAQC)?

To address this question, we used data on the operational and promotional costs associated with the NYSSQL. Operational costs refer to the amount of money NYSDOH spends on NYSSQL each year. Operational costs for NYSSQL include both a fixed component and a variable, per person or per service, costs. For simplicity, and because more detailed quitline cost data were not available to us, we created a measure of average operational cost per quitline client that includes both the fixed and variable costs of operating NYSSQL. Paid anti-tobacco media that features or promotes the quitline telephone number in the advertisements has been shown to effectively promote calls to quitlines [4,18,19,20]. New York has used paid media, including television, radio, print, Internet, and other avenues, consistently and successfully to promote and prompt smokers to contact NYSSQL [21]. To illustrate potential promotional costs associated with increasing the quitline reach to recommended levels, we used data on how much NYSDOH spends each year on paid television media advertisements. Most of NYSDOH’s paid television advertisements promoted tobacco use cessation and also featured the NYSSQL telephone number. Our measure of promotional costs is limited to television advertisements. In practice, a variety of different promotional efforts, including both paid and non-paid, could be used to promote the quitline and increase quitline utilization and reach. Depending on what mix of promotion is used, and the effectiveness of those different types of promotion on prompting smokers to call the quitline, the additional promotional costs may vary from our estimates. These estimates illustrate the additional promotional effort and costs necessary to substantially increase the average annual quitline reach. These estimates are not intended for predictive purposes.

We calculated the total additional paid television media costs and quitline operational costs needed to service the number of quitline clients required to achieve the two recommended levels of reach—8% and 16%. To do this, we scaled the actual annual costs of paid television media and quitline operations in 2016 by the ratio of the hypothetical vs. actual number of NYSSQL clients receiving services in 2016. We then calculated annual marginal paid television media and quitline operational costs by subtracting actual annual costs in 2016 from our estimated 2016 annual costs. We calculated all cost estimates separately for simulation results based on the 7-month follow-up survey responder rate and intent-to-treat variations of the 6-month quit rates.

## 3. Results

Research Question 1: What is the current impact of quitlines on population-level smoking prevalence?

**Figure 1:**
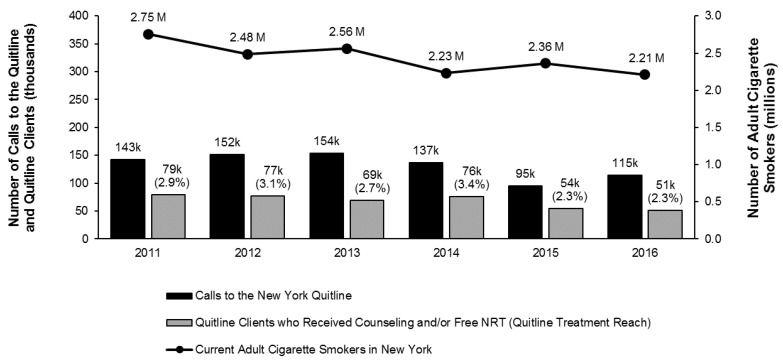
Number of current adult cigarette smokers in New York, calls to the New York State Smokers’ Quitline (NYSSQL), and NYSSQL Clients who received counseling and/or free nicotine replacement therapy (NRT), 2011–2016; Notes: quitline treatment reach is defined as the percentage of adult cigarette smokers who registered for quitline service and received counseling and/or free NRT. Quitline treatment reach values included in the figure are rounded to the nearest tenth; reach was 2.30% in 2015 and 2.32% in 2016.

The total number of current adult cigarette smokers in New York declined from 2.75 million in 2011 to 2.21 million in 2016. During this period, calls to the NYSSQL averaged around 132,600 per year and ranged from 95,000 in 2015 to 154,000 in 2013. Approximately 67,700 tobacco users received counseling and/or free NRT from the NYSSQL each year from 2011 through 2016, with a range from 51,000 in 2016 to 79,000 in 2011. Quitline reach, which is the annual proportion of current adult cigarette smokers in New York who received counseling and/or free NRT from the NYSSQL, averaged 2.78% from 2011 through 2016 and ranged from 2.30% in 2015 and 2016 to 3.40% in 2014 (Figure 1). Based on data from CDC’s NQDW, New York’s annual state quitline reach was ranked between the second and fifth highest among 45 states that reported data for all quarters from 2011 through 2016 (https://www.cdc.gov/statesystem/).

**Figure 2:**
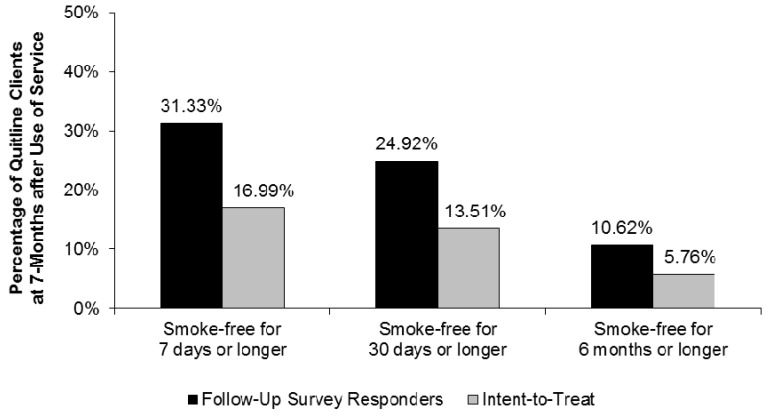
Quit status at 7 months after receipt of New York State Smokers’ Quitline (NYSSQL) Services, 7-month follow-up evaluation survey data, 2011–2014. Notes: follow-up survey responders’ rates are among individuals who completed a 7-month follow-up evaluation survey. Intent-to-treat rates are among all individuals who were sampled for and contacted to complete 7-month follow-up evaluations, regardless of whether they completed the evaluation. This measure attempts to control for nonresponse bias by assuming that all individuals who were contacted for but did not complete a 7-month follow-up evaluation failed to remain smoke-free.

From June 2010 through December 2014, seven-month follow-up evaluation surveys were completed by 7842 individuals among a random sample of 14,461 NYSSQL clients, yielding a total survey response rate of 54.23%. Among these survey respondents, 31.33% were smoke-free for seven days or longer, 24.92% for 30 days or longer, and 10.62% for six months or longer at seven months after receiving quitline services (Figure 2). Using the intent-to-treat approach, an estimated 16.99% of NYSSQL clients sampled for seven-month follow-up evaluation surveys were smoke-free for seven days or longer, 13.51% for 30 days or longer, and 5.76% for six months or longer at seven months after receiving quitline services.

Approximately 51,200 tobacco users received counseling and/or free NRT from the NYSSQL in 2016 (Table 1). The number of those individuals expected to be smoke-free for six months or more at seven months after receipt of services from the quitline ranges from 2900 (5.76%) to 5400 (10.62%) depending on whether the intent-to-treat quit rate or the follow-up survey responder quit rate is used. The prevalence of current adult cigarette smoking in New York was 14.20% in 2016 (Table 1). Removing the number of estimated quitline quitters in 2016 from the adult cigarette smoking population, assuming those individuals do not relapse and can be considered permanent quitters, would reduce the prevalence of current adult cigarette smoking in New York to between 14.16% and 14.18%. These results suggest that, at its current level of reach, the NYSSQL could be responsible for absolute annual reductions in current statewide adult cigarette smoking of 0.02 to 0.04 percentage points each year.

Research Question 2: What would be the impact on population-level smoking prevalence if quitline reach increased to recommended levels (CDC Best Practices for Comprehensive Tobacco Control, NAQC)?

In the scenario that the NYSSQL achieves the 8% reach recommended in CDC’s *Best Practices for Comprehensive Tobacco Control*, the quitline would have provided counseling and/or free NRT to 177,000 quitline clients in 2016 (Table 2). Using the six-month intent-to-treat and responder quit rates to estimate quitline clients’ quit outcomes, the NYSSQL would have generated between 10,200 to 18,800 quitters if it reached 8% of New York’s adult smoker population in 2016. Under this scenario, New York’s prevalence in 2016 would have decreased from 0.07 to 0.12 percentage points, from 14.20% to between 14.08% and 14.13%.

In the second simulated counterfactual scenario, the NYSSQL would have provided counseling and/or free NRT to 354,000 quitline clients in 2016 if it attained the NAQC’s recommended reach of 16% (Table 2). Based on the six-month intent-to-treat and responder quit rates, the NYSSQL would have induced between 20,400 and 37,600 tobacco users to quit in 2016. Under this scenario, New York’s prevalence in 2016 would have decreased from 0.13 to 0.24 percentage points, from 14.20% to between 13.96% and 14.07%.

Research Question 3: How much would it cost to increase annual quitline reach to the levels recommended in CDC’s Best Practices for Comprehensive Tobacco Control and by the NAQC?

In 2016, NYSDOH spent approximately $5.77 million on television media (Table 2). Most of these advertisements promoted tobacco use cessation and featured the NYSSQL telephone number. The total cost of operating the NYSSQL was $2.94 million in 2016. Assuming a direct correspondence between current and proposed media expenditures and inflating these costs by the ratio of hypothetical quitline clients to actual quitline clients, we estimate about $20 million in annual paid television media expenditures would be required to drive enough tobacco users to the NYSSQL and achieve the 8% reach recommended in CDC’s *Best Practices for Comprehensive Tobacco Control*. Approximately $39.81 million in annual television media expenditures would be needed to drive enough tobacco users to the NYSSQL and achieve the NAQC’s recommended reach of 16%. The annual operational costs for the quitline would be about $10.20 million to attain a reach of 8% and $20.31 million to attain a reach of 16%.

## 4. Discussion

Results from this study suggest that at current levels of quitline reach, state quitlines have a low impact on population-level smoking prevalence. From 2011 through 2016, the NYSSQL had an average annual reach of 2.9%, which was nearly three times higher than the national average, making New York one of the top five states in the United States in terms of state quitline reach [15]. Despite its high reach relative to other states and comparable quit rates [15], the NYSSQL is responsible for estimated reductions in population-level smoking rates of only 0.02 to 0.04 percentage points per year. If NYSSQL achieved the recommended annual reach of 8% (CDC Best Practices) and 16% (NAQC), state-level prevalence would decrease by about 0.07–0.12 and 0.13–0.24 percentage points per year, respectively. This means NYSSQL would need to provide cessation services to about three to seven times the number of smokers it served in 2016. In 2016, NYSDOH spent $2.94 million, which was 7% of NY TCP’s annual budget of $39.3 million, on the NYSSQL. Assuming operational costs increased proportionally with increased numbers of smokers receiving services from NYSSQL, NYSDOH would need to spend between $7 million and $17 million annually on NYSSQL operational costs. This would account for approximately 26% to 52% of NY TCP’s annual budget.

We would not expect substantial changes or increases in annual quitline reach without additional promotional efforts. Many factors influence the quitline reach, such as paid and earned media, other promotional efforts, referrals from health care providers, word of mouth, and previous use of the service. Among these, promotional efforts, including paid media, have shown increased quitline calls, and paid media is one of the primary methods used by states and the federal government to promote quitlines and increase quitline use. Previous studies have shown a significant relationship between exposure to paid cessation-focused media that directly promotes quitlines or features the quitline telephone number and calls to quitlines [19,20,21,22,23,24]. For this study, we did not estimate the promotional efforts necessary to attain recommended levels of reach, nor did we cost out specific promotional efforts that might be utilized. However, any successful promotional efforts would have significant financial cost to the state. For illustrative purposes, we calculated a crude estimate of those costs based on a proportional extrapolation of current state tobacco control program expenditures on paid television media, which includes ads that focus on promoting tobacco use cessation and feature the quitline number. In 2016, NYSDOH spent approximately $5.77 million on paid television media, which represented about 15% of the NY TCP’s annual budget. If paid media must increase by three to seven-fold to increase the annual number of NYSSQL clients by three to seven times, then annual paid television media costs would increase between $14 million and $34 million. These increased annual paid television media costs would account for about 51% to 102% of the annual NY TCP budget.

Although quitlines are recommended as an evidence-based part of comprehensive TCPs and provide direct services to smokers that can effectively help increase their chances of quit success, quitlines do not and probably cannot have a substantial impact on statewide smoking prevalence. Quitline reach is too low to result in population-level changes in overall smoking prevalence. Furthermore, achieving a level of reach sufficient to have a population-level impact on smoking prevalence is likely not possible. Our results indicate that quitline reach may not be scalable for several reasons. There may be natural limits on attainable quitline reach due to smokers’ propensity or willingness to use quitlines. Should states achieve the dramatic increases in quitline reach necessary to reach recommended levels, the operational costs of providing quitline services to that number of smokers would likely be cost prohibitive for many states. It is unlikely that reach would spontaneously increase without further deliberate attempts to promote and encourage the use of quitlines. Many states already invest heavily in promotional efforts, and federal promotional efforts also promote quitlines. To achieve substantial increases in reach beyond current levels, the additional promotional expenditures would likely fall outside the range of available state and federal media and promotional budgets. Finally, there is no guarantee that additional promotional efforts will lead to continued increases in reach due to natural limits in the number and percentage of smokers who are receptive to using quitline services. If states wish to increase the utilization of quitline services through promotional efforts and mass media, additional formative research may be needed to identify effective ways to do that, which would also require additional expenditures and resources.

Our results suggest it might be necessary to reconsider the stated role of quitlines in tobacco control efforts. Because states have limited funding and resources for tobacco control efforts, they must invest their limited resources in programs and interventions that will reach the largest number of tobacco users and have the greatest effectiveness at reducing population-level tobacco use. Quitlines may be better suited to serve as support mechanisms to other programs, interventions, or tobacco control efforts. In New York, quitlines are being integrated into a larger initiative to promote cessation through health systems change efforts that use, (1) regional contractors who target medical and behavioral health system administrators to educate about the need to incorporate tobacco dependence treatment into workflow routine, and (2) paid media messaging to encourage smokers to ask for and expect smoking cessation assistance from their health care provider. Previous research has shown that the rate of quitting without assistance is around 2% to 3% in the United States and that brief cessation advice from a health care provider can increase quit rates by an additional 1% to 3% [25]. Nearly 80% of smokers in the United States see a primary care physician each year, with fewer than 50% of those smokers reporting that they received brief advice to quit from their physician [26,27]. The estimated quit rates for quitline callers in New York ranged between 5.76% and 10.62%, which is higher than the estimated 3% to 6% quit rate with brief advice to quit. However, the proportion of smokers in the United States who receive brief advice to quit from a health care provider each year is substantially larger than the annual proportion of smokers who receive counseling services from quitlines. As such, the health care system has a significantly larger reach and larger potential for influencing population-level smoking rates. In New York, NYSSQL is increasingly being seen as an “extender” and promoter of provider-assisted quitting, a more likely way to reach large numbers of smokers and have a population-level impact. Quitline callers are now given more information about insurance coverage of cessation resources and are strongly encouraged to follow-up with their health care providers regarding cessation counseling and medications. NYSSQL is increasingly expected to interact with providers, who are ultimately in a better position to provide ongoing cessation treatment.

## 5. Limitations

This study has several limitations. First, our estimates of NYSSQL callers who had quit for six months or longer at seven months after receiving quitline services were based on data from seven-month follow-up evaluation surveys conducted among a random sample of quitline clients between June 2010 and December 2014. Quit rates are based on survey respondent data. To correct for likely survey nonresponse bias, we use quit rates based on an intent-to-treat approach that assumes all individuals who were sampled for but did not complete follow-up evaluation surveys failed to remain smoke-free. We use both versions of the six-month quit rates and present all results as a range based on these two quit rates. Second, we assume that quitline callers who were smoke-free for six months or longer at the seven-month follow-up were permanently quit and did not relapse. Third, we assume that individuals who use the quitline would not have quit otherwise. Quitlines operate in an environment where other factors, including tobacco control program activities and efforts, are encouraging them to quit. Determining the independent influence of the quitline aside from those other factors is difficult. Our assessment likely overstates the influence of the quitline on tobacco use behavior change, as some of the quitline clients may have used multiple forms of cessation assistance or quit anyway, even without using the quitline. Fourth, our annual estimates of the number of quitline callers who received counseling services from the New York quitline were compiled based on summing quarterly quitline utilization data. If individuals received counseling services from the quitline during more than one quarter during a year, then our estimates of the total number of unique callers who received counseling services, as well as our estimates of annual quitline reach, will be slightly overstated. Fifth, based on previously published research, we assume the effects of paid television media featuring the quitline telephone number on quitline calls are linear. However, at higher levels of paid media implementation, the relationship between paid media and quitline calls may not be linear. At some point, diminishing marginal returns may set in, and additional media will not continue to produce the same proportional increases in quitline calls. This suggests that our estimates of the resources needed to achieve a particular level of reach are likely underestimated. Fifth, we examined promotional costs associated with only paid television media. There are other ways to promote quitlines, including unpaid approaches. Our assessment oversimplifies the promotional efforts and associated costs with increased promotion of quitline services. Sixth, we have presented paid media as primarily being used to promote quitlines and to encourage tobacco users to call quitlines. The purpose of paid media is broader than just promoting quitlines and has been shown to also effectively promote independent, unassisted, quitting behaviors by tobacco users. Finally, our calculations assume all adult cigarette smokers in New York have an equal willingness to quit and propensity to use quitline services. Some adult smokers currently have no desire or willingness to quit, and certain individuals or subpopulations are less interested in calling a quitline and using quitline services. After the quitline reaches smokers who are ready to quit or are receptive to using quitline services, it will be increasingly difficult to get smokers who are not ready to quit or have a lower general propensity for using quitline services to call a quitline. None of these limitations negate our findings or conclusions that, at current levels of reach, state quitlines have a limited maximum potential impact on overall population-level smoking prevalence and increasing reach beyond current levels would be difficult and likely cost-prohibitive for many states.

## 6. Conclusions

Although quitlines are recommended as an evidence-based part of comprehensive tobacco control programs, and they provide effective services to individuals who call, quitline reach is too low to result in population-level changes in overall smoking prevalence. Increasing quitline reach may not be feasible and would likely be cost-prohibitive. Because states have limited funding and resources for tobacco control efforts, they must invest their limited resources in programs and interventions that will reach the largest number of tobacco users and have the greatest effectiveness at reducing population-level tobacco use. Our results suggest it might be necessary to re-think the role of quitlines in tobacco control efforts. Quitlines may be better suited to serve as support mechanisms to other programs, interventions, or tobacco control efforts. In New York, the quitline is being integrated into larger efforts to promote cessation through health systems change. A substantially higher proportion of smokers see a health care provider each year compared to the proportion of smokers who use quitline services. As such, the health care system has a significantly higher reach and greater potential for influencing population-level smoking rates.

## Figures and Tables

**Table 1:** Estimated impact of the New York State Smokers’ Quitline (NYSSQL) on adult cigarette smoking prevalence in New York, 2016.

**Estimation Inputs/Parameters**	**Follow-Up Survey Respondents**	**Intent-to-Treat**
Total adult population in New York (Census)	15,565,000	15,565,000
Prevalence of current adult cigarette smoking (BRFSS)	14.20%	14.20%
Number of current adult cigarette smokers	2,210,000	2,210,000
Annual number of quitline clients who received counseling and/or free NRT	51,200	51,200
Quitline quit rate: percentage of quitline clients smoke-free for 6-months or longer at 7 months after use of quitline services	10.62%	5.76%
**Estimation Results**	**Follow-Up Survey Respondents**	**Intent-to-Treat**
Quitline quitters: number of quitline clients smoke-free for 6 months or longer at 7 months after use of quitline services	5400	2900
Current adult cigarette smokers minus quitline quitters	2,204,600	2,207,100
State-level quit rate associated with quitline quitters	0.24%	0.13%
Estimated prevalence of current adult cigarette smoking associated with quitline quitters	14.16%	14.18%
Estimated absolute change in annual state-level smoking prevalence associated with quitline quitters	−0.04%	−0.02%

Notes: Follow-up survey responders’ rates are among individuals who completed a 7-month follow-up evaluation survey. Intent-to-treat rates are among all individuals who were sampled for and contacted to complete 7-month follow-up evaluations, regardless of whether they completed the evaluation. This measure attempts to control for nonresponse bias by assuming that all individuals who were contacted for but did not complete a 7-month follow-up evaluation failed to remain smoke-free.

**Table 2:** Simulation results for different levels of annual quitline reach, 2016.

Measure	Actual NY Quitline Reach	CDC Best Practices Recommended Reach (8%)	NAQC Recommended Reach (16%)
Annual quitline reach	2.3%	8.0%	16.0%
Annual quitline clients receiving counseling and/or free NRT	51,200	177,000	354,000
Ratio of simulated quitline clients to actual quitline clients	1.0	3.5	6.9
Annual television media costs	$5.77 million	$20.00 million	$39.81 million
Annual quitline operational costs	$2.94 million	$10.20 million	$20.31 million
Total annual New York Tobacco Control Program (NY TCP) budget	$39.30 million	$39.30 million	$39.30 million
Annual television media costs as a percentage of total NY TCP budget	15%	51%	102%
Annual quitline operational costs as a percentage of Total NY TCP budget	7%	26%	52%
Quitline quit rate: Percentage of quitline clients smoke-free for 6-months months or longer at 7-months after use of quitline services	10.62% [RR] 5.76% [ITT]	10.62% [RR] 5.76% [ITT]	10.62% [RR] 5.76% [ITT]
Quitline quitters: Number of quitline clients smoke-free for 6 months or longer at 7-months after use of QL services	5400 [RR] 2900 [ITT]	18,800 [RR] 10,200 [ITT]	37,600 [RR] 20,400 [ITT]
Current adult cigarette smokers minus quitline quitters	2,204,600 [RR] 2,207,100 [ITT]	2,191,200 [RR] 2,199,800 [ITT]	2,172,400 [RR] 2,189,600 [ITT]
State-level quit rate associated with quitline quitters	0.24% [RR] 0.13% [ITT]	0.85% [RR] 0.46% [ITT]	1.70% [RR] 0.92% [ITT]
Estimated statewide prevalence of current adult cigarette smoking (Actual statewide current adult smokers minus quitline quitters)	14.16% [RR] 14.18% [ITT]	14.08% [RR] 14.13 [ITT]	13.96% [RR] 14.07% [ITT]
Absolute reduction in statewide adult smoking prevalence associated with quitline quitters	−0.04% [RR] −0.02% [ITT]	−0.12% [RR] −0.07% [ITT]	−0.24% [RR] −0.13% [ITT]

Notes: Follow-up survey responders’ rates (RR) are among individuals who completed a 7-month follow-up evaluation survey. Intent-to-treat rates (ITT) are among all individuals who were sampled for and contacted to complete 7-month follow-up evaluations, regardless of whether they completed the evaluation. This measure attempts to control for nonresponse bias by assuming that all individuals who were contacted for but did not complete a 7-month follow-up evaluation failed to remain smoke-free.
